# Uvaol attenuates TGF-β1-induced epithelial-mesenchymal transition in human alveolar epithelial cells by modulating expression and membrane localization of β-catenin

**DOI:** 10.3389/fphar.2024.1504556

**Published:** 2025-01-07

**Authors:** Liliane Patrícia Gonçalves Tenório, Felipe Henrique da Cunha Xavier, Mônica Silveira Wagner, Kayo Moreira Bagri, Erick Gabriel Alves Ferreira, Romulo Galvani, Claudia Mermelstein, Adriana Cesar Bonomo, Wilson Savino, Emiliano Barreto

**Affiliations:** ^1^ Cell Biology Laboratory, Federal University of Alagoas, Maceió, Brazil; ^2^ Laboratory on Thymus Research, Oswaldo Cruz Institute, Oswaldo Cruz Foundation, Rio de Janeiro, Brazil; ^3^ National Institute of Science and Technology on Neuroimmunomodulation, Oswaldo Cruz Institute, Oswaldo Cruz Foundation, Rio de Janeiro, Brazil; ^4^ Rio de Janeiro Network on Neuroinflammation, Oswaldo Cruz Institute, Oswaldo Cruz Foundation, Rio de Janeiro, Brazil; ^5^ INOVA-IOC Network on Neuroimmunomodulation, Oswaldo Cruz Institute, Oswaldo Cruz Foundation, Rio de Janeiro, Brazil; ^6^ Cell Structure and Dynamics Laboratory, National Cancer Institute, Rio de Janeiro, Brazil; ^7^ Muscle Differentiation Laboratory, Institute of Biomedical Sciences, Federal University of Rio de Janeiro, Rio de Janeiro, Brazil

**Keywords:** uvaol, TGF-β1, epithelial-mesenchymal transition, β-catenin, cell migration

## Abstract

The epithelial-mesenchymal transition (EMT) is a biological process in which epithelial cells change into mesenchymal cells with fibroblast-like characteristics. EMT plays a crucial role in the progression of fibrosis. Classical inducers associated with the maintenance of EMT, such as TGF-β1, have become targets of several anti-EMT therapeutic strategies. Natural products from the pentacyclic triterpene class have emerged as promising elements in inhibiting EMT. Uvaol is a pentacyclic triterpene found in olive trees (*Olea europaea* L.) known for its anti-inflammatory, antioxidant, and antiproliferative properties. Yet, its effect on the TGF-β1-induced EMT in alveolar epithelial cells is unknown. The present study aimed to investigate the impact of uvaol upon TGF-β1-induced EMT in a cultured A549 human alveolar epithelial cell line, a classic *in vitro* model for studies of EMT. Changes in cell shape were measured using phase-contrast and confocal microscopy, whereas protein expression levels were measured using immunofluorescence, flow cytometry, and Western blotting. We also performed wound scratch experiments to explore its effects on cell migration. Uvaol had no significant cytotoxic effects on A549 cells. By contrast, the changes in the cell morphology consistent with TGF-β1-induced EMT were largely suppressed by treatment with uvaol. In addition, increased contents of mesenchymal markers, namely, vimentin, N-cadherin, and fibronectin in TGF-β1-induced A549 cells, were downregulated by uvaol treatment. Furthermore, the TGF-β1-induced migration of A549 cells was significantly suppressed by uvaol. Mechanistically, uvaol prevented the nuclear translocation of β-catenin and reduced the TGF-β1-induced levels of ZEB1 in A549 cells. These results provide compelling evidence that uvaol inhibits EMT by regulating proteins related to the mesenchymal profile in human alveolar epithelial cells, likely by modulating β-catenin and ZEB1 levels.

## 1 Introduction

The epithelial-mesenchymal transition (EMT) is the process wherein immotile epithelial cells lose their polarity and epithelial characteristics while acquiring mesenchymal phenotypes and behaviors. This transition occurs under certain physiological or pathological conditions, such as fibrosis (Dongre and Weinberg, 2018). The molecular and biochemical hallmarks of EMT include reduced expression of epithelial markers, such as E-cadherin and cytokeratins, along with increased expression of mesenchymal markers, such as N-cadherin and vimentin. These changes are accompanied by cytoskeletal reorganization, enhanced extracellular matrix (ECM) protein production, altered expression of adhesion molecules, and activation of various transcription factors (Loh et al., 2019; Syed et al., 2023).

The transforming growth factor beta-1 (TGF-β1) signaling pathway is the most well-characterized mechanism driving EMT. TGF-β1 induces EMT through multiple intracellular signaling cascades. While canonical TGF-β1/Smad signaling is the primary mechanism underlying EMT induction, non-canonical (non-Smad) pathways, including those mediated by Ras/MAPK, PI3K/Akt, and β-catenin also play significant roles (Datta et al., 2021). Among these, the β-catenin signaling pathway has been strongly implicated in the pathogenesis and progression of tissue fibrosis (Akhmetshina et al., 2012). The interplay between β-catenin and TGF-β1 signaling contributes to ECM deposition in mesenchymal-derived cells across various organs (Maarouf et al., 2016). Studies have highlighted the involvement of β-catenin signaling in both tissue repair and fibrosis, demonstrating its regulatory role in the differentiation of mesenchymal stem cells into specific cell types (Cao et al., 2018). Furthermore, genetic disruption of β-catenin transcription has been shown to effectively mitigate kidney fibrotic lesions (Hao et al., 2011). These findings underscore the critical role of β-catenin signaling, particularly following TGF-β1 receptor activation in epithelial cells, as a pathogenic mechanism driving tissue fibrosis.

Several studies suggests that natural compounds can modulate the signaling pathways implicated in EMT (Park et al., 2015). Compounds such as resveratrol and curcumin have been reported to influence both canonical and non-canonical TGF-β1 pathways during EMT (Kandagalla et al., 2022; Song et al., 2019). These findings highlight the potential of natural-derived agents as therapeutic candidates for targeting EMT-associated process and conditions, including fibrosis.

Uvaol (Urs-12-ene-3,28-diol) is a natural pentacyclic triterpenoid found in virgin olive oils, as well as wide variety of fruits, vegetables, and medicinal plants. It exhibits numerous pharmacological effects, including anticancer, antioxidant, and anti-inflammatory activities (Sánchez-Quesada et al., 2013). Uvaol has demonstrated beneficial effects in various pathological conditions and is characterized by low toxicity (Agra et al., 2016; Carmo et al., 2020), making it a promising candidate for potential clinical therapies. Previous studies have shown that uvaol alleviates angiotensin II-induced cardiac hypertrophy and left ventricular remodeling in rats by reducing fibrosis in the myocyte area (Martín et al., 2012). Similarly, uvaol has been reported to protect against cardiac fibrosis by downregulating α-SMA, collagen, and TGF-β1 expression (Siddesha et al., 2014; Wang et al., 2017). Additionally, plant-derived natural products like uvaol represent an important source for drug discovery and development (Li et al., 2022), offering a high cost-benefit ratio compared to synthetically or biologically designed drugs, which are often more expensive (Domingo-Fernández et al., 2024). The absence of cytotoxicity, combined with the effect of uvaol to suppress chronic inflammation, inhibit myofibroblast activation, and reduce extracellular matrix accumulation, supports its potential as a modulator of biological process related to tissue repair and fibrosis, such as EMT. Despite these promising attributes, the effects and underlying mechanism of uvaol in reducing tissue fibrosis remains to be elucidated. In this study, we employed an *in vitro* model of TGF-β1-induced EMT in human alveolar A549 cells to investigate the protective role of uvaol in EMT and the potential mechanism involved.

## 2 Material and methods

### 2.1 Reagents

The following chemicals (purchased from Sigma Chemical Co., St. Louis, MO, United States), were used: uvaol (Urs-12-ene-3,28-diol, ≥95% purity, PubChem CID: 92802), Dulbecco’s Modified Eagle Medium high glucose (DMEM), phosphate-buffered saline (PBS), antibiotic antimycotic solution (×100), fetal calf serum (FCS), trypsin, Triton X-100, L-glutamine, 4′,6-diamidino-2′-phenylindole dihydrochloride (DAPI), bovine serum albumin (BSA), tris-buffered saline (TBS), Tween-20, sodium deoxycholate, β-mercaptoethanol, mitomycin C and dimethyl sulfoxide (DMSO). Phalloidin-Alexa fluor 633™ and anti-GAPDH were purchased from Santa Cruz Biotechnology (California, United States). Stock solution of uvaol was prepared in DMSO. The DMSO concentration applied in cell cultures never exceeded 0.1%. Neither the vehicle nor any compounds used in this study altered cell viability. Recombinant human TGF-β1 was purchased from PeproTech (New Jersey, United States).

### 2.2 Cell culture

Type II alveolar epithelial cells, derived from a human adenocarcinoma (A549 cells) were obtained from the Rio de Janeiro Cell Bank (https://bcrj.org.br). The cells were cultured in high glucose DMEM supplemented with 10% FCS and antibiotic antimycotic solution (×1) and maintained at 37°C until reaching 80% confluence in a 5% CO_2_ atmosphere before treatments and stimulations.

### 2.3 Cell viability assessment

The effect of uvaol on cell viability was measured by flow cytometry using the Fixable Viability Stain^®^ 510 kit (BV510, BD Biosciences, San Diego, United States) to discriminate living from unviable cells. Briefly, A549 cells (5 × 10^4^ cells/well) were seeded into 24-well plates and then incubated in serum-free medium with different concentrations of uvaol (2.5, 5, 10, 20, and 40 µM) for 48 h. After this period, the cells were removed with cold PBS-EDTA solution 0.2 g/L, washed twice with cold PBS, resuspended, and incubated for 7 min at 37°C with reagent 1:200 diluted Fixable Viability Stain (FVS) in 2% FBS. The acquisition was performed using a FACS Celesta flow cytometer (BD Biosciences, California, United States), and data were analyzed using the FlowJo™ v10.8 software (BD Biosciences). 50,000 events were acquired for each sample to perform the appropriate statistical treatment.

### 2.4 Epithelial-mesenchymal transition induction and treatments

A549 cells were treated with varying concentrations of TGF-β1 (2.5 ng/mL, 5 ng/mL, or 10 ng/mL) or serum-free medium alone for 48 h. The cells were then fixed with cold absolute methanol for 5 min and stained using a rapid panoptic kit. Morphological observations were made using inverted phase contrast microscopy (Olympus CKX31, Tokyo, Japan). In another set of experiments, cells were pre-treated for 1 h with 10 µM uvaol or serum-free medium (untreated controls). The culture medium was removed, and another aliquot of uvaol (2.5 µM or 10 μM, Supplementary Material) was added, in the presence or absence of TGF-β1 (5 ng/mL), in a serum-free medium.

### 2.5 Immunostaining and cell morphology analysis

A549 cells (6 × 10^3^ cells/well) were seeded in an 8-well Lab-Tek^®^ Chamber Slide™ plate and subjected to uvaol treatment for 48 h with or without TGF-β1 (5 ng/mL) or serum-free media alone. Next, the cells were fixed with 4% paraformaldehyde in PBS for 20 min or cold absolute methanol for 10 min. After fixation, the cells were washed twice with PBS and permeabilized with 0.05% Triton X-100 in PBS (v/v) for 15 min, followed by blocking with 1% BSA and 8% FCS in PBS/Triton-X100 for 40 min. For F-actin labelling, cells were incubated with phalloidin-Alexa fluor 633™ (diluted 1:100) for 30 min. In other wells, cells were incubated for 1 h with primary anti-pan-cytokeratin, anti-vimentin, anti-pan-cadherin, anti-E-cadherin, anti-β-catenin, or anti-fibronectin antibodies (see Table 1). Subsequently, the specimens were washed three times with PBS and then incubated with appropriate secondary antibody (Alexa Fluor^®^ 594 goat anti-rabbit immunoglobulin IgG or Alexa Fluor^®^ 488 goat anti-mouse IgG; Invitrogen Corporation), followed by DNA staining with 4′,6-diamidino-2-phenylindole (DAPI; blue fluorescence) for 10 min at room temperature. The slides were washed twice with PBS and mounted in Prolong gold™ medium (Invitrogen, France). Labeling was evaluated using fluorescence microscopy (AxioImager A2—AxioVision Rel 4.8 software, Zeiss, Germany). Negative controls included the presence of the secondary antibody alone and the use of PBS/Triton only. Cells stained with phalloidin were evaluated under a laser confocal microscope (Leica TSC-SP8 Confocal Laser Scanning Microscope, Wetzler, Germany), and images were acquired using LAS-X Software 3.1 (Leica Microsystems). Morphological parameters such as “*circularity*,” “*roundness*,” and “*aspect ratio*” were quantitated using the ImageJ Fiji software (Zanier et al., 2015).

**TABLE 1:** General features of antibodies applied in the study.

Antibody	Species	Company	Catalogue number	Dilution (IF^a^)	Dilution (WB^a^)
Anti-pan-cytokeratin	Human, Mouse	DAKO	M3515	1:25	1:2000
Anti-vimentin	Human	Abcam	Ab16700	1:100	1:1,000
Anti-E-cadherin	Human	BD Bioscience	610182	1:250	1:1,000
Anti-fibronectin	Human, mouse	DAKO	A0245	1:100	—
Anti-β-catenin	Human, mouse	Invitrogen	138400	1:250	—
Anti-CD49e	Human	Abcam		1/100	
Anti-GAPDH	Human	--	--	--	1:50,000

Antibody	Fluorochrome	Species	Company	Catalogue number	Dilution (FWC^a^)
Anti-CD49e	BV421	Human	BD Bioscience	740077	1:100
Anti-CD49d	BV480	Human	BD Bioscience	566134	1:20
Anti-N-cadherin	BV605	Human	BD Bioscience	740432	1:20
Anti-CD49f	BV650	Human	BD Bioscience	563707	1:100
Anti-CD29	BB515	Human	BD Bioscience	564565	1:100
Anti-Vimentin	PE	Human	BD Bioscience	562337	1:50
Anti-N-cadherin	Percep-Cy5.5	Human	BD Bioscience	563435	1:20

^a^ IF, immunofluorescence; WB, western blotting; FWC, flow cytometry.

### 2.6 Western blotting

Cells (6 × 10^5^/well) were seeded in 25 cm^2^ flasks and subjected to uvaol treatment for 48 h with or without TGF-β1 (5 ng/mL) or serum-free media and lysed for 1 h on ice using 300 mL of lysis buffer (50 mM Tris-HCL in pH 7.5, 150 mM NaCl, 1 mM EDTA-pH 8.0, 1% NP-40, 0.5% sodium doxycholate, and 0.1% SDS). Lysates were loaded into SDS-polyacrylamide gels (SDS-PAGE) after being denatured by heating for 5 min at 95°C while using β-mercaptoethanol. The proteins were then transferred onto nitrocellulose blotting membranes (GE Healthcare, Chicago, United States). The membranes were blocked for 1 h at room temperature with 5% non-fat dried milk in 0.1% TBS-Tween-20 before being incubated with various primary antibodies detailed in Table 1. The membranes were then incubated for 1 h at room temperature with rabbit or mouse HRP-conjugated secondary antibodies, followed by chemiluminescent detection with an enhanced chemiluminescence kit (Thermo Scientific™ Pierce™ ECL Western blotting substrate, Waltham, MA, United States). Protein bands were quantified using a chemiluminescence system and normalized to respective GAPDH (Bio-Rad ChemiDoc™ Imaging System, California, United States).

### 2.7 Flow cytometry

For flow cytometry analyses, A549 cells (1.5 × 10^5^/well) were seeded in 24-well plates and treated with uvaol for 48 h in the presence and absence of TGF-β1 or serum-free medium. Cells were then gently detached using a cold PBS-EDTA solution (0.20 g/L), being subsequently fixed with 1 × solution fixation/permeabilization concentrate (eBioscience, San Diego, United States) at 4°C for 1 h, washed twice, and incubated with undiluted permeabilization buffer (eBioscience, San Diego, United States) for 15 min at 4°C. Nonspecific sites were blocked with a 1% BSA and 8% FCS solution using a 1 × permeabilization buffer for 40 min. After blocking, cells were incubated with a cocktail of fluorochrome-conjugated monoclonal antibodies (anti-CD29, anti-CD49d, and CD49e, as detailed in Table 1) for 1 h at 4°C, followed by washing with undiluted permeabilization buffer. Analyses were performed using a flow cytometer (FACS Celesta) coupled to the FACSDiva™ Software v9.0 (BD-Biosciences, San Diego, United States) and analyzed using the FlowJo™ Software v10.8 (BD-Biosciences, San Diego, United States). Fluorescence minus one (FMO) control measurements were used for setting cut-off threshold “gates” identification of the cell population positive versus negative events, beads for positive control and unlabeled cells incubated only with buffer as negative control (Fox et al., 2020).

### 2.8 Scratch wound healing assay

Twenty thousand A549 cells per well were seeded into 24-well plates and maintained until reaching 90% confluence. Cells were treated with 3 μg/mL mitomycin C (Sigma-Aldritch) for 3 h. The monolayers were gently and slowly scratched with a 200 μL pipette tip across the center of the well. After washed with PBS twice, cells were treated or not with TGF-β1 alone (5 ng/mL) or in combination with 10 µM uvaol for 24 h and 48 h. Images were taken by an Olympus CKX31 microscope coupled to a digital camera at 0, 24, and 48 h. Cell migration was analyzed using the ImageJ software, and gap closure was expressed as a percentage (%) of the control. The value was obtained through the following calculation: (%) = [(A0- At)/A0] × 100. A0 is the area at time 0, and At is the area after the gap.

$$\frac{Wound\;closure\;\%=\left[{Α}_{t=oh}-{Α}_{t=}{∆}_{h}\;\right]\;x\;100\%\;}{\;{Α}_{t=oh}}$$

### 2.9 Statistical analyses

Data were expressed as mean ± standard error of the mean (SEM). The Kolmogorov-Smirnov normality test was applied to determine the normal distribution between samples in the morphometric analysis. Then, we used the one-way variance test (ANOVA) followed by Tukey’s multiple-comparison test. Differences in which *p* ≤ 0.05 were considered statistically significant. The studies were carried out using GraphPad Prism^®^ software version 8.01.

## 3 Results

### 3.1 Uvaol does not affect cell viability in cultured A549 epithelial cells

**FIGURE 1:**
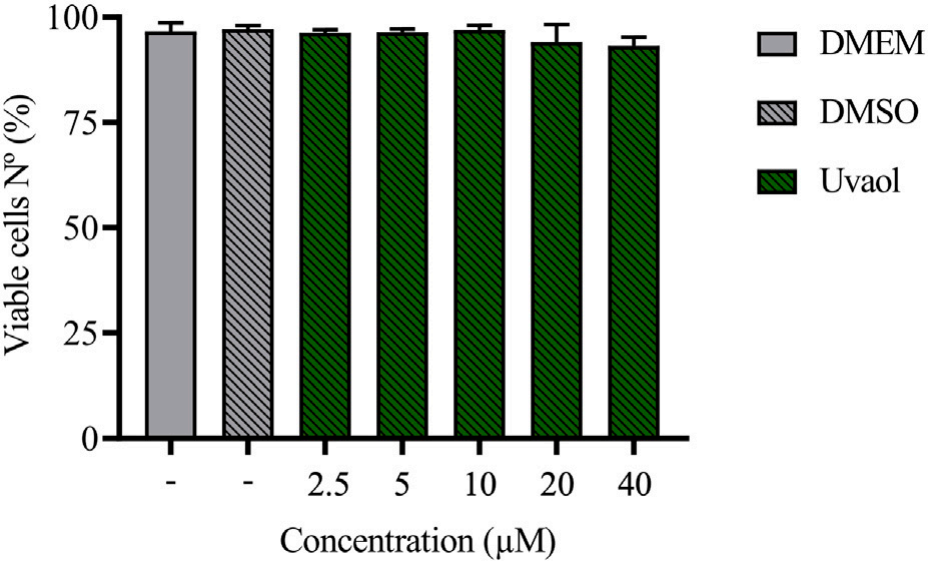
Treatment with uvaol does not alter the viability of A549 cells. Cells were treated with uvaol (2.5–40 µM), vehicle (0.01% DMSO) or culture medium alone (DMEM) for 48 h. Cell viability was determined by FVS labelling and analysed by flow cytometry. Bar graphs represent mean values ± S.E.M.

The effect of uvaol on cell viability was assessed using flow cytometry to exclude toxicity. Across all tested concentrations, uvaol demonstrated no cytotoxicity toward A549 cells, as determined by the FVS viability assay after 48 h of treatment (Figure 1; Supplementary Figure S1). These findings align with previous studies that reported the absence of uvaol toxicity in other cell types (Bonel-Pérez et al., 2020; Carmo et al., 2020). Given that no tested concentration of uvaol affected cell viability, we selected concentrations of 2.5 µM and 10 µM to evaluate the optimal threshold for effect of uvaol on TGF-β1-induced EMT. While 2.5 µM uvaol did not significant impact the morphological changes associated with TGF-β1-induced EMT (Supplementary Figure S2), the 10 μM concentration showed pronounced effects. Therefore, we used 10 μM uvaol in subsequent experiments. Supporting this choice, prior studies have shown that 10 μM uvaol effectively modulates oxidative stress and exerts anti-inflammatory effects *in vitro* (Allouche et al., 2011; Silva et al., 2021). With this concentration established, we next investigated the effect of TGF-β1-mediated changes on the morphology of A549 cells.

### 3.2 TGF-β1 causes A549 alveolar epithelial cells to shift to a mesenchymal phenotype

**FIGURE 2:**
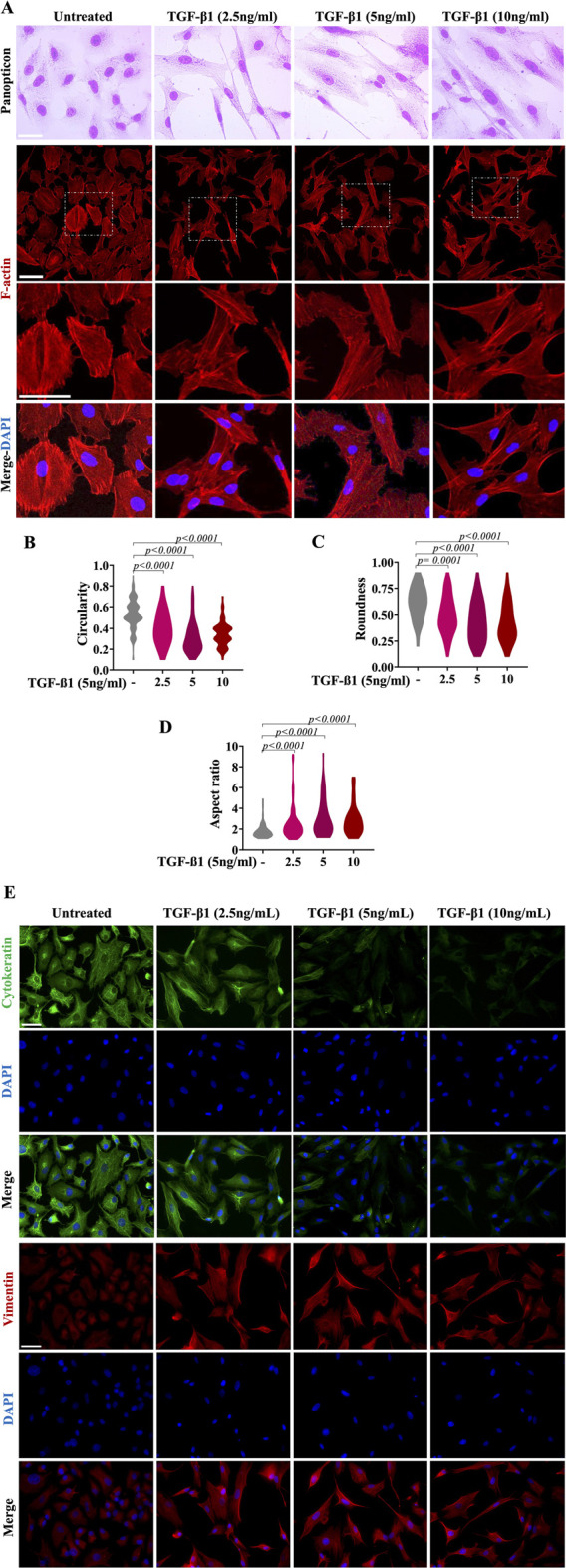
TGF-β1 induces morphological and molecular changes in A549 alveolar epithelial cells. Morphologic changes consistent with EMT were observed in TGF-β1-treated cells for 48 h, compared with those untreated cells (control, cells that were not exposed to TGF-β1, cultured with medium DMEM) maintained the cobblestone morphology characteristic of epithelial cells. The phenotypic changes (transition towards a fibroblast-like phenotype) in TGF-β1-treated cells were compared to untreated cells and evaluated by light microscopy and panoptic staining [upper micrographs in panel **(A)**]. Lower micrographs revealed phalloidin staining of A549 cells incubated for 48 h as indicated without TGF-β1 (untreated) or treated with TGF-β1 at 2.5 ng/mL, 5 ng/mL or 10 ng/mL Phalloidin (red) was used to detect F-actin, while DAPI (blue) was used to decorate the nuclei. Scale bar, 100 µm. The violin plots reveal the distribution of circularity **(B)**, roundness **(C)** and aspect ratio **(D)** in A549 cells treated with medium (DMEM) alone or TGF-β1 for 48 h. Morphometric analyses from violin plots were performed using ImageJ FiJi software, and statistically significant differences between groups are indicated by One-way ANOVA followed by the Kruskal–Wallis test. *p* values are shown in each graph. Panel **(E)** depicts immunofluorescence analyses of EMT-related intermediate filament proteins in A549 alveolar epithelial cells. Cells show a decrease in cytokeratin and, in parallel, an increase in vimentin after TGF-β1-treatment. Phenotypic marker changes were consistent with EMT in TGF-β1-treated A549 cells for 48 h, compared with those untreated cells (control cells cultured with medium DMEM). DAPI counterstaining was used to detect nuclei (blue). Scale bar = 100 µm.

A549 cells were exposed to different concentrations of TGF-β1 (2.5 ng/mL, 5 ng/mL and 10 ng/mL) and morphologically evaluated 48 h later. As expected, the TGF-β1-treated A549 cells showed elongation, branching, and the loss of the cobblestone-like appearance (Figure 2A). Untreated cells (not exposed to TGF-β1) maintained the cobblestone morphology characteristic of epithelial cells (Figure 2A). Moreover, untreated cells exhibited cortical actin staining adjacent to the cell membrane, whereas the TGF-β1-treated cells displayed elongated F-actin stress fibres (Figure 2A). These changes in cell morphology were confirmed by analysis of cell shape descriptors. We observed a significant reduction in cell circularity and roundness parameters, accompanied by increased cell aspect ratio in all TGF-β1-treated cell conditions after 48 h (Figures 2B–D). These findings collectively denote features consistent with elongated and spindle-shaped cellular morphology, as supported by observations under phase-contrast microscopy and significant alterations in the actin cytoskeleton.

We further examined by immunofluorescence the expression of typical epithelial markers, including cytokeratin, and one typical mesenchymal marker, vimentin, in the presence and absence of TGF-β1 in A549 cells. As shown in Figure 2E, before TGF-β1 treatment, cytokeratin was intensely expressed in A549 cells, whereas vimentin showed a very low expression, being somewhat restricted to the perinuclear region. After treatment with increasing TGF-β1 concentrations, there was a dose-dependent reduction in cytokeratin contents in A549 cells. We observed that, at TGF-β1 concentrations of 5 and 10 ng/mL, the protein levels of this epithelial marker were virtually abolished in almost all cells. Regarding vimentin, before treatment, it was weakly expressed in A549 cells. However, after TGF-β1 treatment, vimentin was seen, being uniformly distributed throughout the cytoplasm, pointing out the fibroblast shape of the cells, confirming the EMT phenotype when compared to the pattern seen in cells that did not receive TGF-β1.

### 3.3 Uvaol attenuated the TGF-β1-induced cell morphological and phenotypic changes of EMT in A549 cells

**FIGURE 3:**
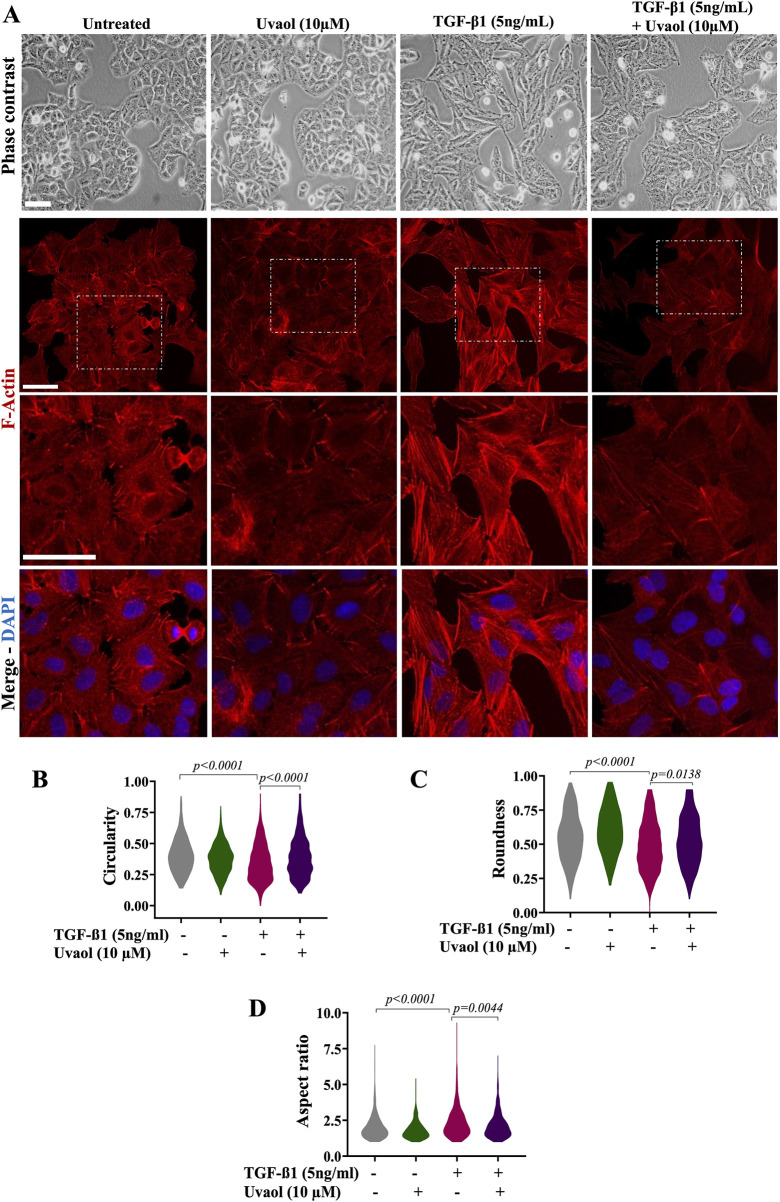
Uvaol prevents morphological changes induced by TGF-β1 on A549 alveolar epithelial cells. Bright-field microscopy showing the effect of uvaol (10 µM) on morphologic changes consistent with EMT in TGF-β1-stimulated A549 cells for 48 h. Untreated cells were those not exposed to TGF-β1 cultured with DMEM. In panel **(A)**, phalloidin staining of A549 cells was incubated for 48 h as indicated without TGF-β1 (untreated) or treated with uvaol (10 µM) and stimulated with TGF-β1 (5 ng/mL). Phalloidin (red) was used to detect F-actin, while DAPI (blue) was used to decorate nuclei. Scale bar, 100 µm. The violin plots illustrate the morphometric parameters, namely, circularity **(B)**, roundness **(C)** and aspect ratio **(D)** in A549 cells treated with medium (DMEM) or uvaol and stimulated with TGF-β1 for 48 h. Morphometric analyses of violin plots were performed using ImageJ FiJi software, and statistically significant differences between groups are indicated by One-way ANOVA followed by the Kruskal–Wallis test. *p* values are shown in each graph.

We next assessed the effect of uvaol upon the TGF-β1-mediated changes in cell morphology in A549 cells. To accomplish this, A549 cells were exposed to uvaol at a concentration of 10 µM in the presence or absence of TGF-β1 at 5 ng/mL for 48 h. As depicted in Figure 2, A549 cells underwent typical EMT morphological changes in response to TGF-β1, there was a loss of cell-to-cell contact, and cells acquired a spindle-shaped and fibroblast-like phenotype (Figure 3A). Conversely, treatment with uvaol reduced the morphological changes induced by TGF-β1. We can observe that the cells presented a less fusiform and elongated phenotype compared to the group of cells that received TGF-β1 alone (Figure 3A). In addition, uvaol treatment interfered with the reorganization of F-actin microfilaments induced by TGF-β1, causing a decrease in the formation of stress fibers compared to cells treated with TGF-β1 alone (Figure 3A).

To better characterize the effect of uvaol on morphological changes induced by TGF-β1, we quantified morphological parameters. As expected, TGF-β1 induced a decrease in cell circularity and roundness, alongside an increase in aspect ratio in TGF-β1-treated cells compared to untreated cells (Figures 3B–D). Conversely, treatment with uvaol in the presence of TGF-β1 increased cell circularity and roundness relative to cells when compared with TGF-β1 alone (Figures 3B, C). This observation was corroborated by the aspect ratio parameter, namely, uvaol treatment with TGF-β1 resulted in a decreased aspect ratio (Figure 3D), showing that uvaol prevented the elongated cell profile induced by TGF-β1.

### 3.4 Uvaol inhibits the expression of EMT markers induced by TGF-β1

**FIGURE 4:**
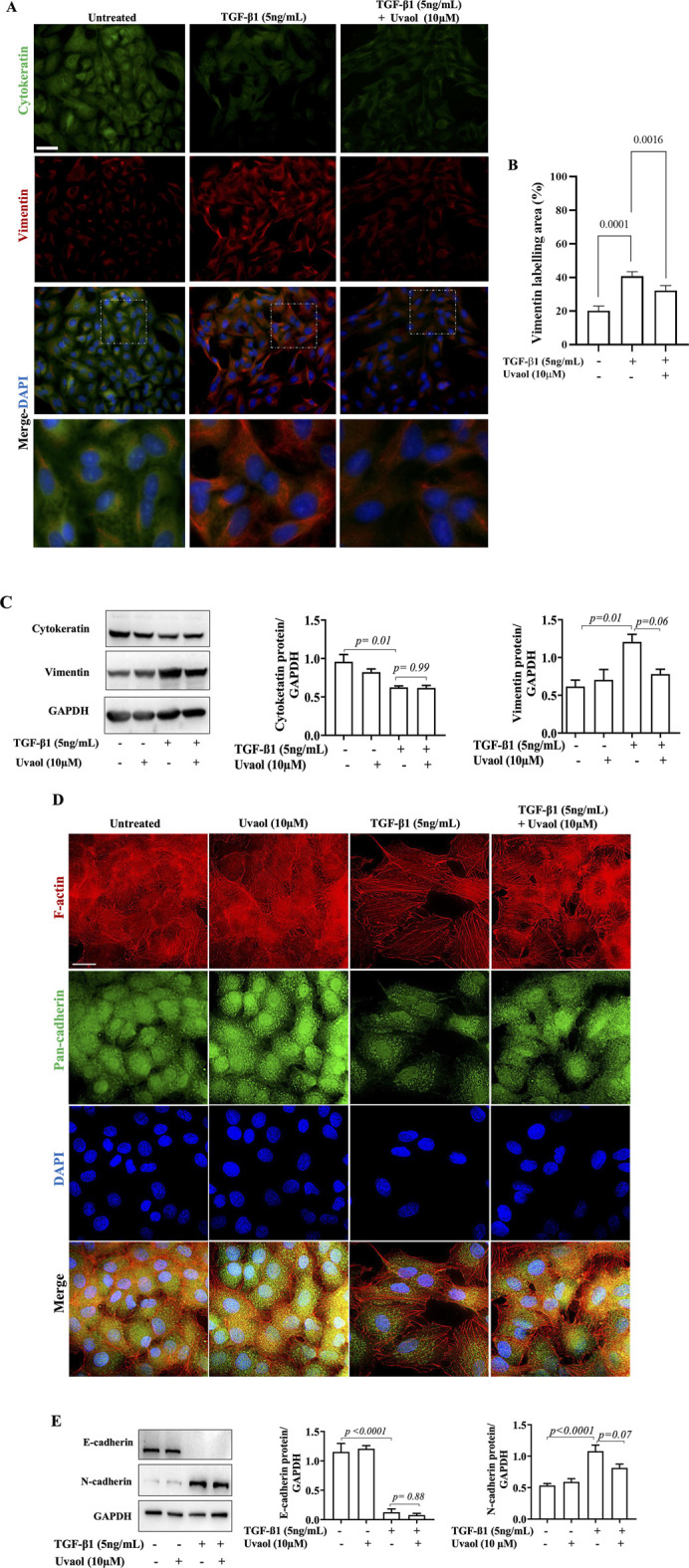
Effect of uvaol on the expression of epithelial-mesenchymal transition biomarkers. A549 cells were treated with TGF-β1 with or without uvaol co-treatment for 48 h. Protein levels of cytokeratin (green) and vimentin (red) was determined by immunofluorescence staining **(A)**. DAPI (blue) was used to detect nuclei. Scale bar: 20 μm. The contents of vimentin was determined by immunofluorescence staining **(B)**. The contents of cytokeratin and vimentin was determined by Western blotting **(C)**. Phalloidin (red) was used to detect F-actin and pan-cadherin (green) was determined by immunofluorescence staining **(D)**. Protein levels of E-cadherin and N-cadherin was determined by Western blotting **(E)**. Quantification of the western blots was performed after 3 independent experiments, and data are expressed as the means ± SEM. Bar graphs show statistical significance between groups, determined by one-way ANOVA followed by Tukey’s post-test. *p* values are shown in each graph.

To further confirm the effects of uvaol on TGF-β1-induced EMT, we analyzed the protein levels of EMT markers. We observed that treatment with TGF-β1 for 48 h induced a significant loss of cytokeratin compared to untreated cells, as revealed by immunofluorescence, and confirmed by Western blot (Figures 4A, C). However, uvaol did not interfere with this change. As expected, TGF-β1 significantly increased vimentin protein levels compared to untreated epithelial cells. Interestingly, uvaol drastically prevented the high expression of vimentin in A549 cells treated with TGF-β1, ascertained by immunofluorescence and Western blotting (Figures 4A, B), being further confirmed by flow cytometry (Supplementary Figure S3).

Changes in cell surface proteins are evident characteristics in EMT. Therefore, we evaluated the effect of uvaol on protein levels of cadherins in TGF-β1-induced EMT. Immunofluorescence showed that TGF-β1 induced a decrease in pan-cadherin immunostaining compared to untreated cells (Figure 4D). Uvaol treatment did inhibit the loss of pan-cadherin levels induced by TGF-β1 (Figure 4D). We further assessed the effect of uvaol on protein levels of E-cadherin and N-cadherin in cells treated with TGF-β1, two key molecules involved in EMT. Western blot analysis revealed a marked decrease in E-cadherin protein levels and an increase in N-cadherin in TGF-β1-treated cells compared to untreated cells, consistent with the typical features of EMT (Figure 4E). Interestingly, uvaol was not able to interfere with the loss of E-cadherin induced by TGF-β1 treatment but did prevent the significant increase in TGF-β1-induced N-cadherin levels (Figure 4E) evaluated by Western blot and confirmed by flow cytometry (Supplementary Figure S3).

### 3.5 Uvaol modulates fibronectin production and VLA-5 receptor expression during TGF-β1-induced EMT in A549 cells

**FIGURE 5:**
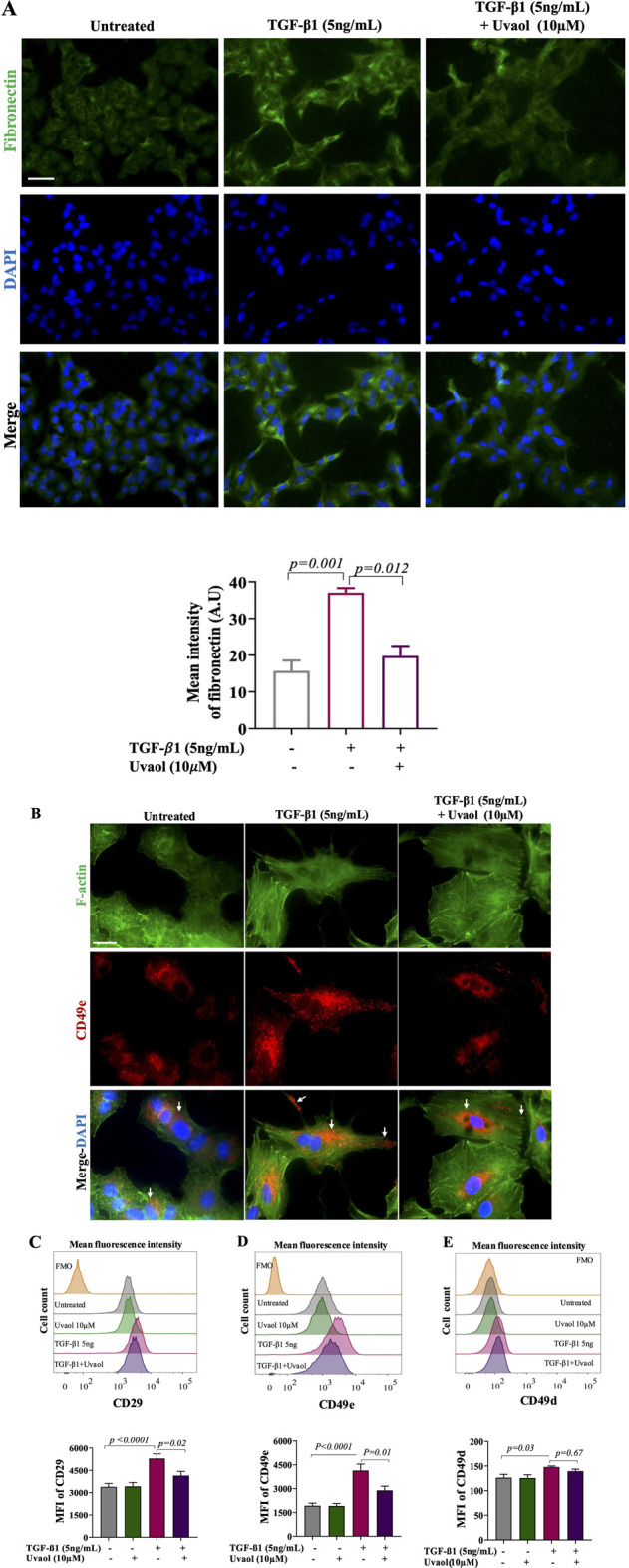
Uvaol modulates fibronectin production and adhesion receptors expression in TGF-β1-stimulated A549 cells. Cells were treated with TGF-β1 with or without uvaol co-treatment for 48 h. Fibronectin contents were ascertained by immunofluorescence staining **(A)**. DAPI (blue) was used to detect nuclei. Scale bar: 100 µm. In **(B)**, phalloidin (green) was used to detect F-actin, and CD49e (red) was identified by immunofluorescence staining (white arrows). Scale bar, 50 µm.The histogram plots and bar charts represent the mean fluorescence intensity (MFI) of integrin receptor subunits CD29 **(C)**, CD49e **(D)** and CD49d **(E)**, determined by flow cytometry. Data are from 4 independent experiments. In the bar graph, data were expressed as the means ± SEM. Bar graphs show statistical significance between groups, determined by one-way ANOVA followed by Tukey’s post-test considering *p* values showed in each graph. AU = arbitrary unit.

Given the critical role of TGF-β1 in EMT induction and the increasing evidence on the involvement of extracellular matrix (ECM) ligands and receptors in this process, we investigated the effect of uvaol on fibronectin production and its receptor VLA-5 (CD49e/CD29) induced by TGF-β1. As shown by immunofluorescence in Figure 5A, TGF-β1-stimulated A549 cells exhibited increased fibronectin levels, as observed by the intensity of immunofluorescence, compared to untreated cells. However, A549 cells co-treated with TGF-β1 and uvaol showed a significant decrease in TGF-β1-induced fibronectin contents. Regarding VLA-5, we observed by immunofluorescence staining and flow cytometry (Figures 5B–D) that TGF-β1 led to a significant increase in CD29 and CD49e subunits, whereas uvaol treatment significantly inhibited TGF-β1-induced protein levels of CD29 and CD49e subunits (Figures 5B–D). No effects of uvaol on CD49d protein levels were observed (Figure 5C). These results suggest that uvaol may exert inhibitory effects on fibronectin production and its respective receptor VLA-5 in TGF-β1-induced EMT.

### 3.6 Uvaol inhibits the loss of β-catenin induced by TGF-β1 at the cell junctions of A549 cells

**FIGURE 6:**
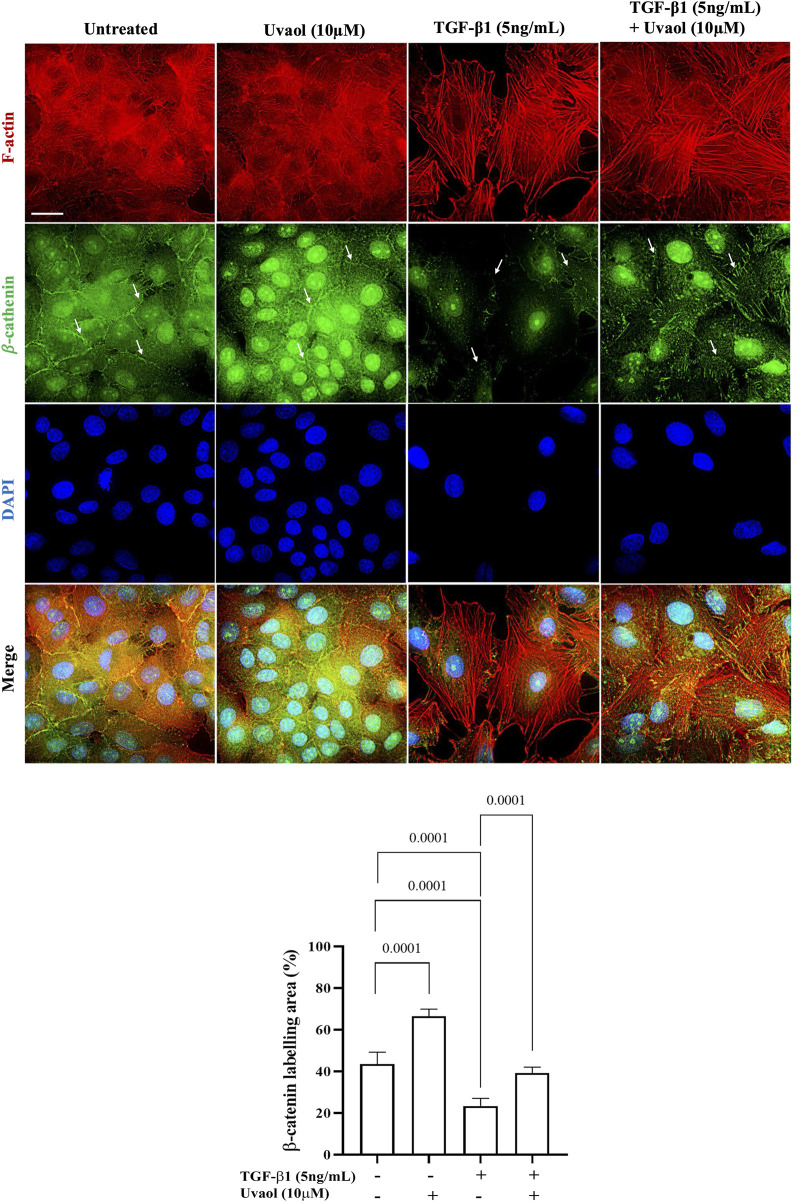
Uvaol attenuates the pronounced loss of β-catenin in the subcellular region adjacent to the plasma membrane, induced by TGF-β1 in A549 cells. Cells were treated with TGF-β1 (5 ng/mL), with or without co-treatment with uvaol, for 48 h. Immunolocalization of β-catenin at adherens junctions (indicated by white arrows) was performed through co-labeling of β-catenin (green) and F-actin (red) in A549 cells. In untreated and uvaol-treated cells, β-catenin is localized adjacent to the plasma membrane and in the nuclear/perinuclear region of A549 cells. In cells treated with TGF-β1 alone, β-catenin at the membrane was drastically lost, primarily localizing in higher concentration in the nuclear/perinuclear region (white arrows) compared to untreated control (DMEM medium). Simultaneous treatment with TGF-β1 and uvaol maintained β-catenin adjacent to the plasma membrane, thereby preventing total loss and exacerbated nuclear translocation of β-catenin induced by TGF-β1. Scale bar: 20 μm.

Overall, the data presented above provide substantial evidence of the inhibitory effects of uvaol on EMT. To investigate the underlying molecular mechanism of uvaol in EMT in A549 cells, we analyzed the localization of β-catenin in cells treated with uvaol and stimulated with TGF-β1. It is widely recognized that β-catenin plays a crucial role in maintaining the stability of adherens junctions, distinctive features of epithelial cells, and acts as the primary signaling molecule in the canonical Wnt pathway, thus contributing to EMT (Guo et al., 2019). As shown in Figure 6, untreated A549 cells exhibited prominent fluorescent staining for β-catenin in the subcellular region adjacent to the cell membrane, essential for maintaining normal epithelial polarity and intercellular adhesions. However, upon stimulation with TGF-β1, a drastic loss of β-catenin at the cell membrane of A549 cells was observed, with its localization predominantly restricted to the nuclear/perinuclear region, compared to untreated cells. Uvaol prevented the pronounced loss of β-catenin at adherens junctions induced by TGF-β1. These results indicate that uvaol modulates the TGF-β1 induced redistribution of β-catenin at the cell membrane, contributing to the maintenance of the epithelial phenotype and reducing the exacerbated nuclear translocation of β-catenin induced by TGF-β1.

### 3.7 Uvaol decreases ZEB1 contents in the nuclear region of A549 cells treated with TGF-β1

**FIGURE 7:**
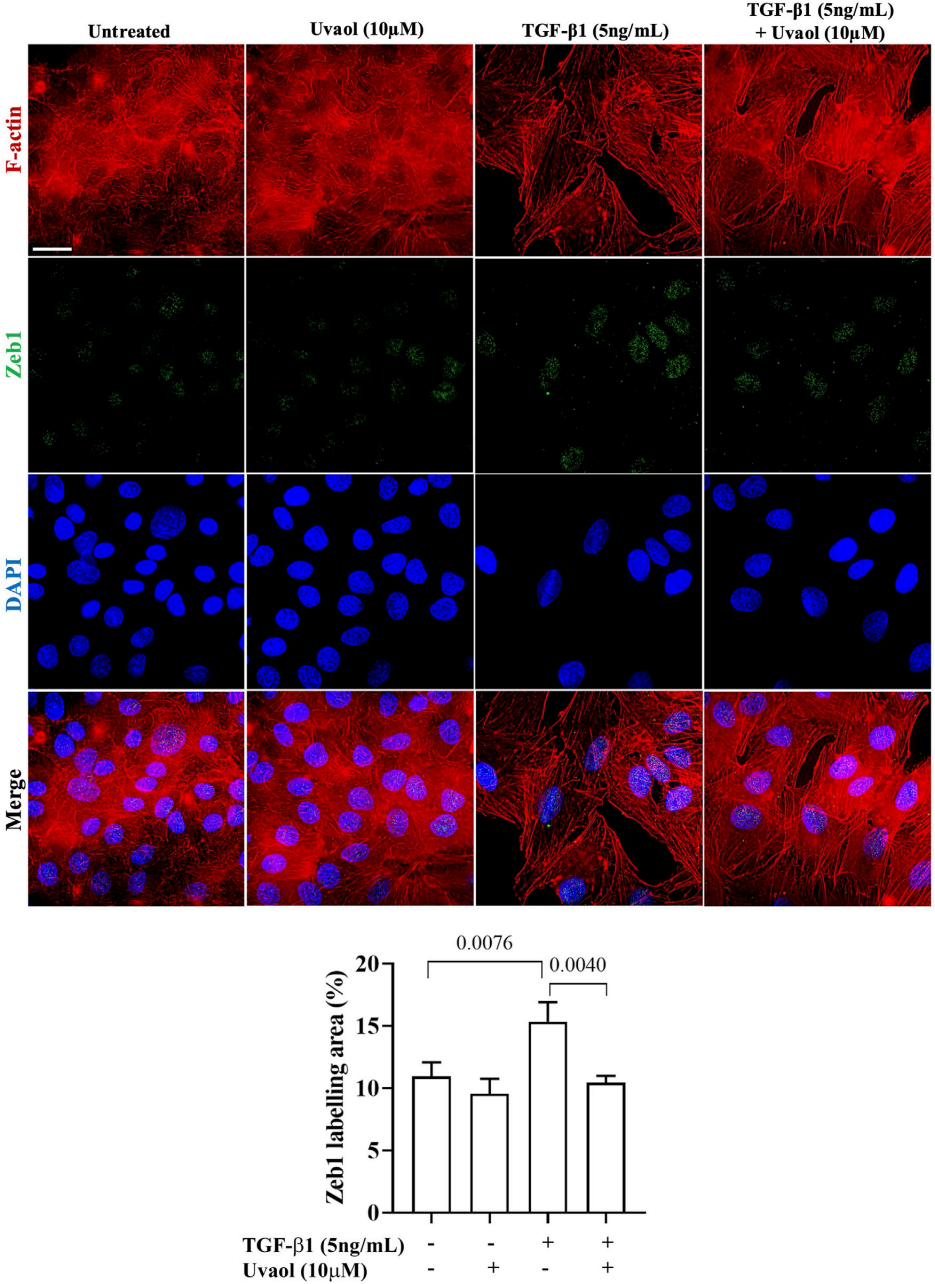
Uvaol decreases ZEB1 contents in the nuclear region of A549 cells treated with TGF-β1. Cells were treated with TGF-β1 (5 ng/mL) with or without uvaol co-treatment for 48 h. Immunolocalization of nuclear/cytoplasmic ZEB1 (indicated by white arrows) was performed by co-labeling ZEB1 (green) and F-actin (red) in A549 cells. Scale bar: 20 µm. DAPI (blue) was used to decorate nuclei. 20 μm. The bar graph shows the total labeling of nuclear/cytoplasmic ZEB1 localization, data were expressed as mean ± SEM. The bar graphs show statistical significance between the groups, determined by one-way ANOVA followed by Tukey’s post-test considering the *p*-values presented in each graph.

1. Nuclear β-catenin can induce the expression of various transcription factors, including ZEB1, one of the main factors responsible for the induction of the EMT process in various pathological contexts (Guo et al., 2019). We then evaluated the effect of uvaol in modulating the nuclear localization of ZEB1 induced by TGF-β1. As shown in Figure 7, A549 cells stimulated with TGF-β1 exhibited a significant increase in nuclear ZEB1 levels compared to untreated cells, whereas treatment with uvaol resulted in a significant reduction in nuclear ZEB1 contents in TGF-β1-induced A549 cells.

### 3.8 Uvaol suppresses TGF-β1-induced migration in A549 cells

**FIGURE 8:**
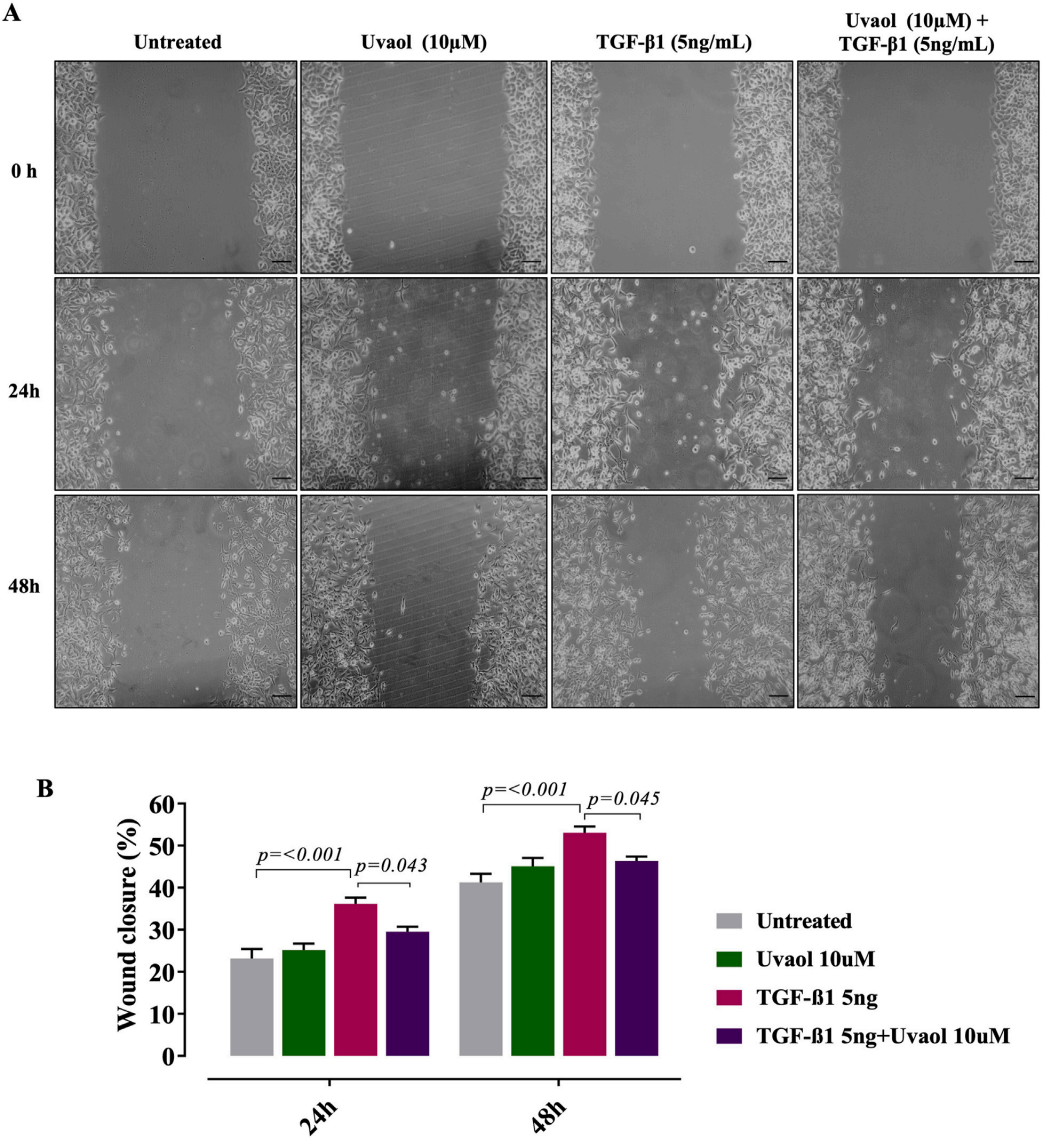
Uvaol prevents the TGF-β1-induced migration of A549 alveolar cells. Cells were treated with TGF-β1 (5 ng/mL) with or without uvaol co-treatment for 24 and 48 h. The migration was measured by the scratch assay. Panel **(A)** depicts representative images of the horizontal migration assay showing the closure of the wound area induced with a tip from pipette on a monolayer of A549 cells and evaluated at 0, 24 and 48 h after treatment with uvaol and stimulation with TGF-β1 (5 ng/mL). In panel **(B)** the bar graphs represent the quantitation of the cell migration at 24 and 48 h after treatment with uvaol or TGF-β1. Statistical significance among groups was determined by one-way ANOVA followed by Tukey’s post-test. *p* values are shown in each graph.

It is well-accepted that the β-catenin signaling pathway plays a central role in inducing EMT, contributing significantly to increased cellular motility (Sánchez-Tilló et al., 2011). In this regard, we assessed the effect of uvaol upon TGF-β1-induced migration using the cell scratch assay. Our results showed that TGF-β1 did increase the number of migrating cells compared to untreated cells (cells maintained with DMEM medium alone) at 24 and 48 h (Figure 8). Conversely, uvaol treatment prevented this effect, consistent with a reduced EMT phenotype in A549 cells (Figures 8A, B).

## 4 Discussion

In this study, we demonstrated that uvaol, a pentacyclic triterpene commonly found in olives and virgin olive oil, markedly inhibits TGF-β1-induced epithelial-mesenchymal transition (EMT) in A549 human alveolar epithelial cells. EMT is a critical process implicated in the pathophysiology of various chronic diseases, including degenerative fibrotic disorders and cancer (Chen et al., 2017; Jolly et al., 2018; Krebs et al., 2017; López-Nouoa and Nieto, 2009). Consistent with previous findings (Yang et al., 2020), we observed that TGF-β1-treated A549 cells lost their characteristic cobblestone morphology and acquired a mesenchymal spindle-like appearance. These morphological changes were accompanied by the suppression of epithelial markers, including cytokeratin, pan-cadherin, and E-cadherin, and an upregulation of mesenchymal markers such as vimentin, and N-cadherin. Remarkably, our results reveal for the first time that uvaol significantly inhibits these morphological and phenotypic changes, highlighting to attenuate TGF-β1-induced EMT in A549 cells. Additionally, we observed that uvaol treatment prevented the TGF-β1-induced increase in fibronectin and its integrin receptor VLA-5. Importantly, uvaol also inhibited the loss of β-catenin from the plasma membrane induced by TGF-β1, preserving signalling pathways critical for maintaining the epithelial phenotype. Moreover, uvaol treatment significantly reduced ZEB1 contents in the nuclear region of TGF-β1-induced A549 cells, suggesting interference with TGF-β1-mediated signalling pathways and subsequent EMT inhibition. Collectively, these findings position uvaol as a promising candidate for modulating EMT-related process, with potential therapeutic implication for fibrotic diseases.

Previous studies have demonstrated that other pentacyclic triterpenoids belonging to the same chemical group as uvaol, specifically those with a ursane-type chemical structure, also exhibit anti-EMT properties. For instance, ursolic acid has been shown to inhibit EMT *in vitro* and *in vivo* by interfering with TGF-β1-induced signalling, suggesting its potential as a therapeutic agent for clinical intervention in renal interstitial fibrosis (Xu et al., 2019). Similarly, asiatic acid, another pentacyclic triterpenoid, suppressed EMT by inhibiting processes such as cell migration, increasing E-cadherin mRNA expression, and reducing the levels of N-cadherin, vimentin, and β-catenin in TGF-β1-treated human alveolar epithelial A549 cells (Cui et al., 2019). These findings position triterpenoids, including uvaol, as a significant class of natural products with potential anti-EMT effects.

It is important to draw attention to the fact that the effects of uvaol were not limited to the morphological aspects of EMT, functional parameters associated with the mesenchymal profile were also affected by treatment with uvaol, as exemplified by the attenuation of TGF-β1-induced migration in A549 cells. EMT is a pivotal process in the initiation and progression of tissue fibrosis and has traditionally been viewed as a mechanism by which epithelial cells transition into fibroblasts, contributing significantly to fibrosis (Kendall and Feghali-Bostwick, 2014). Preventing EMT is thus a viable therapeutic strategy for mitigating tissue fibrosis (Fintha et al., 2019).

Several natural compounds have shown promising pharmacological activity against EMT and its mediators (Anwar et al., 2022). Previous studies demonstrated uvaol’s anti-cancer effects through modulation of PI3K/Akt and JAK/STAT pathways (Bonel-Pérez et al., 2020) and its protective effects against acute lung inflammation and gut damage by inhibiting inflammatory responses (Martín et al., 2009a). Similarly, uvaol has been shown to prevent inflammation and oxidative stress in endothelial and trophoblast cells *in vitro* (Silva et al., 2021). However, its potential to inhibit EMT was previously unclear. In present study, cytological analysis revealed that uvaol effectively reduced morphometrical changes associated with TGF-β1-induced EMT, including circularity and roundness, which are linked to actin reorganization and stress fiber formation. Cytoskeletal reorganization is critical for EMT, enabling epithelial cells to acquire mesenchymal properties (Carvalho Leão et al., 2023; Li and Wang, 2020). Supporting our results, uvaol was previously reported to inhibit actin stress fiber formation in human astrocytoma cells (Martín et al., 2009b) and reduce cytoskeletal changes in an *in vitro* model of chorioamnionitis (Botelho et al., 2019).

In accordance with previous studies, TGF-β1 induced mesenchymal markers (N-cadherin and vimentin) and suppressed epithelial markers (cytokeratin and E-cadherin). In our study, we showed that uvaol significantly inhibited TGF-β1-induced increases in N-cadherin and vimentin levels but had no effect on E-cadherin. This selective modulation suggests that uvaol may partially influence hallmark EMT parameters. Since partial EMT is increasingly recognized as a key driver of metastatic cancer cell dissemination (Pastorino et al., 2024), further investigation is warranted to clarify this phenomenon. These findings align with studies demonstrating that pentacyclic triterpenes inhibit EMT markers *in vivo* and *in vitro* (Loh et al., 2019; Venkataswamy et al., 2023; Xu et al., 2019; Zhang et al., 2015). Additionally, uvaol reversed the TGF-β1-induced increase in fibronectin and its integrin receptor α5β1 (VLA-5), which are known to play crucial roles in fibrosis and cell-matrix adhesion (Schnittert et al., 2018). Overexpression of α5β1 integrin has been reported in human lung myofibroblasts from Idiopathic pulmonary fibrosis (IPF) patients and is associated with EMT in renal fibrosis models and cancer cell migration (Erdogan et al., 2017; Shochet et al., 2020; White et al., 2007). Moreover, consistent with reports of others pentacyclic triterpenes, uvaol also inhibited TGF-β1-induced migration of colorectal cancer (Wang et al., 2019; Zhang et al., 2015) and hepatocarcinoma cells (Bonel-Pérez et al., 2020). EMT-driven migration involves β-catenin translocation to the nucleus, activating transcription of genes related to cell motility (Liu et al., 2022). Nuclear β-catenin activates transcription factors like ZEB1, which suppress epithelial markers and increase mesenchymal gene expression, thereby enhancing EMT (Guo et al., 2019; Sánchez-Tilló et al., 2011).

Our results revealed that uvaol drastically inhibited TGF-β1-induced β-catenin loss from the cytoplasmic membrane and reduced nuclear ZEB1 levels, effectively mitigating mesenchymal phenotype and migration. Since ZEB1 is a direct β-catenin target gene (Sánchez-Tilló et al., 2011), we propose that uvaol’s suppression of β-catenin nuclear translocation contributes to ZEB1 downregulation. Interestingly, uvaol increased β-catenin localization at cell junctions in untreated and TGF-β1-treated cells, supporting its role in maintaining epithelial integrity.

In conclusion, our study highlights the potential uvaol as a natural compound to inhibit TGF-β1-induced EMT in A549 cells. By preventing β-catenin translocation and modulating EMT markers, uvaol emerges as a promising therapeutic candidate for diseases characterized by tissue remodeling and fibrosis. However, further studies are essential to elucidate its precise molecular mechanisms and validate its efficacy *in vivo*.

## Data Availability

The original contributions presented in the study are included in the article/Supplementary Material, further inquiries can be directed to the corresponding authors.
