# Supplementary material for: Uvaol attenuates TGF-β1-induced epithelial-mesenchymal transition in human alveolar epithelial cells by modulating expression and membrane localization of β-catenin
Source: Front Pharmacol. 2025 Jan 7;15:1504556. doi: 10.3389/fphar.2024.1504556 (PMC11747490; doi:10.3389/fphar.2024.1504556)
Supplement: Supplementary file 1 [file DataSheet1.docx]

Supplementary Material

**Manuscript title:** Uvaol attenuates TGF-β1-induced epithelial-mesenchymal transition in human alveolar epithelial cells by modulating expression and membrane localization of β-catenin

**Authors:** Liliane Patrícia Gonçalves Tenório, Felipe Henrique da Cunha Xavier, Mônica Silveira Wagner, Kayo Moreira Bagri, Erick Gabriel Alves Ferreira, Romulo Galvani, Claudia Mermelstein, Adriana Cesar Bonomo, Wilson Savino, Emiliano Barreto

## Supplementary Figure 1


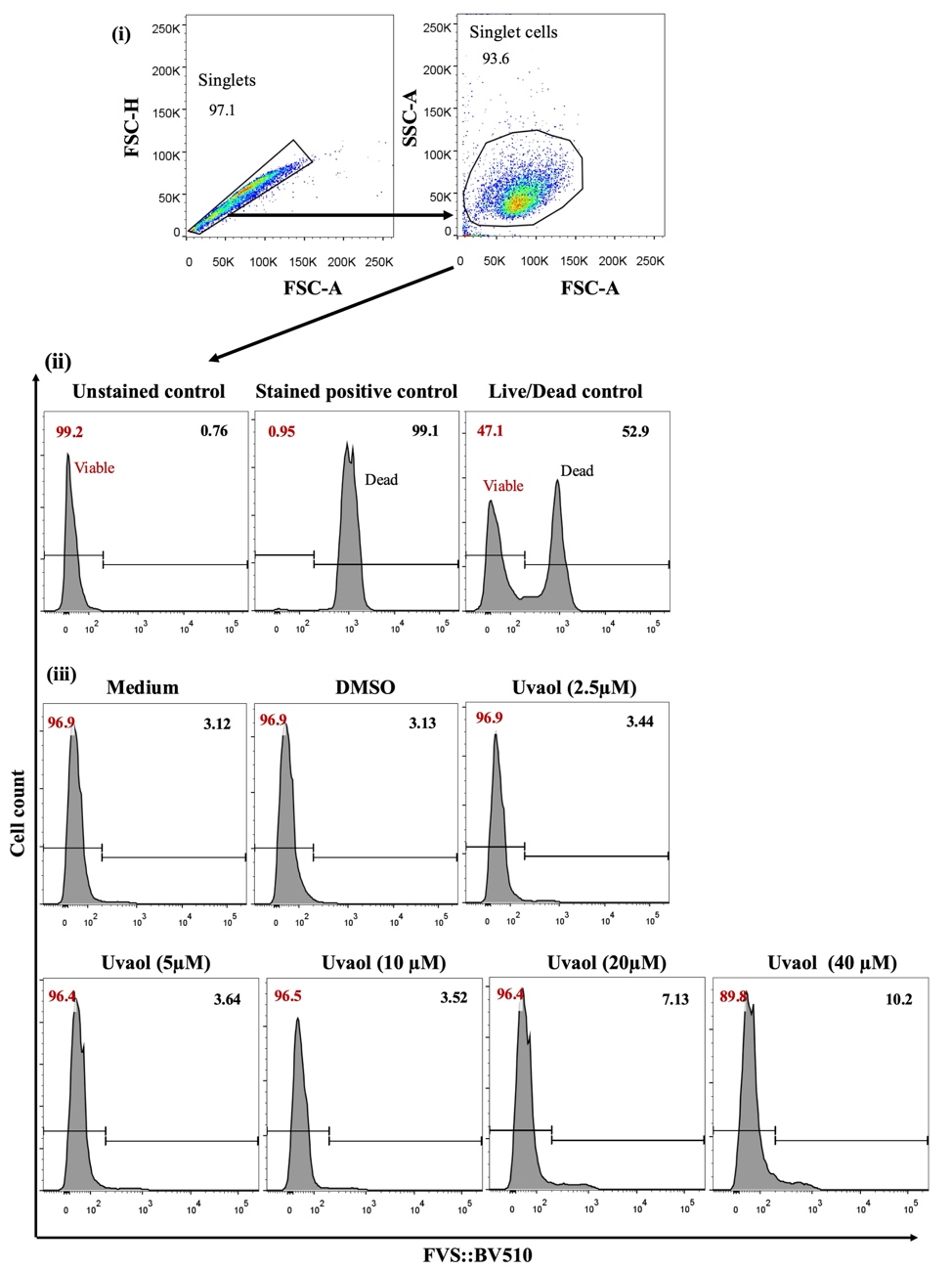


**Supplementary Figure 1.** Gate strategy employed for flow cytometric analysis in A549 cells treated with varying concentrations of uvaol (2.5 µM, 5 µM, 10 µM, 20 µM, or 40 µM). **(i)** The gating strategy for singlets is illustrated on a scatter plot of forward scatter height (FSC-H) versus forward scatter area (FSC-A). Subsequently (indicated by a black arrow), for singlet cells, we applied a gating strategy using side scatter area (SSC-A) versus FSC-A. **(ii, iii)**. For the identification of viable (red numbers) and non-viable (black numbers) cell populations, we used a histogram gating strategy of SSC-A versus Fixable Viability Stain 510 (FVS-510). In **(ii)**, the histogram representing the frequency of viable (red) and non-viable (black) cells in the unstained control, stained control, and live/dead control, used to assess the differential profile (the population of living cells was easily distinguished from the dead population). The unstained control was employed to define the gates to identify the living versus cell populations. In **(iii)**, the number in the histograms represents the frequency of viable (red) and non-viable (black) cell numbers in the untreated groups (medium and DMSO) and those treated with different concentrations of uvaol.

## Supplementary Figure 2


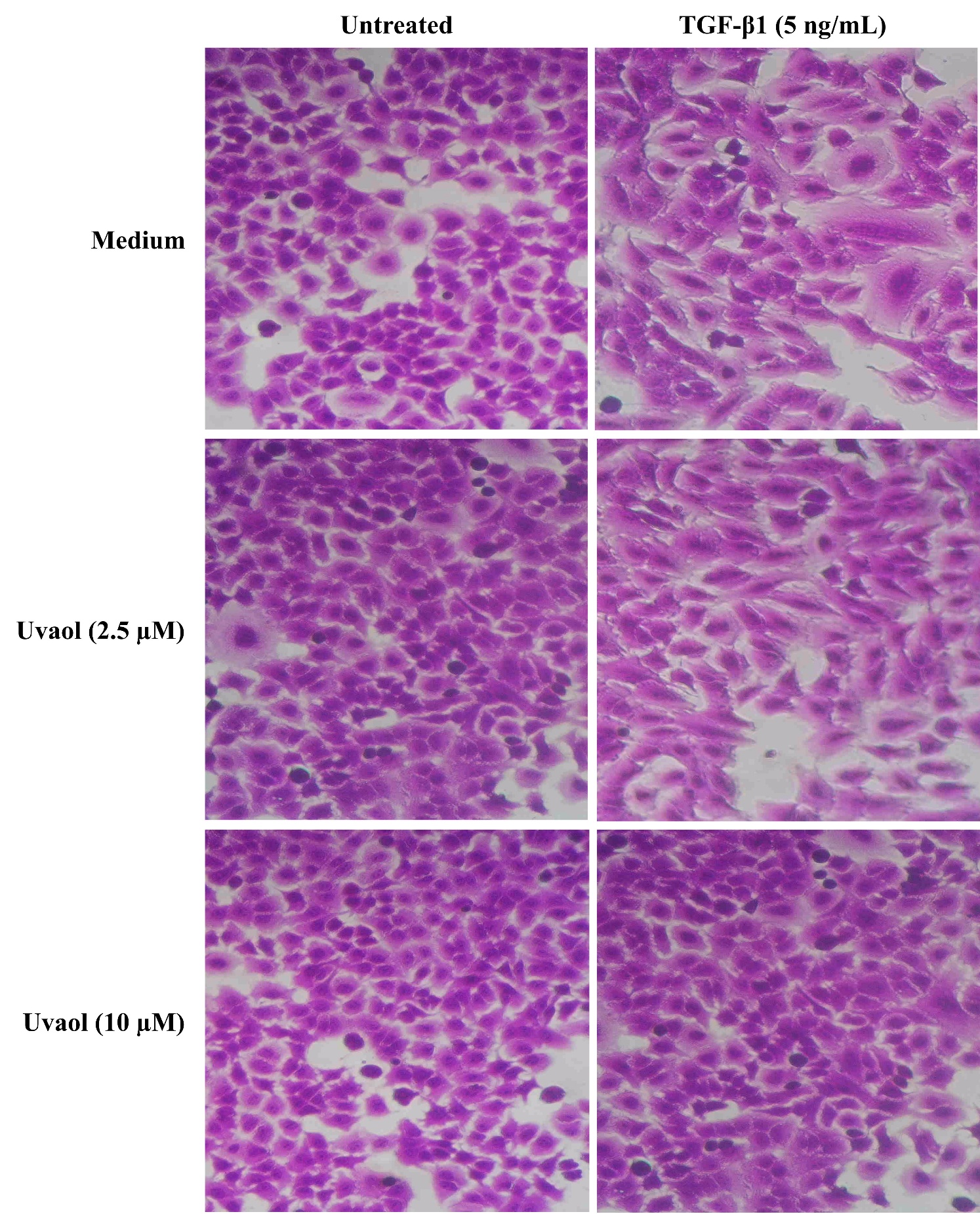


**Supplementary Figure 2.** Effect of uvaol on the TGF-β1-induced epithelial to mesenchymal transition (EMT). The cells were stimulated with TGF-β1 (5 ng/mL) in presence or absence of uvaol at two concentration (2.5 µM or 10 µM) for 48 hours. Following treatment, cells were fixed with cold absolute methanol for 5 minutes and stained with crystal violet for morphological evaluation. Photographs were taken at 48 hours. Morphological changes were observed using phase contrast microscope at a magnification of 100×. Upon TGF-β1 treatment, cells displayed a characteristic shift in morphology from the typical cobblestone epithelial cells shape to an elongated, spined-like mesenchymal phenotype. In contrast, cells co-treated with TGF-β1 and uvaol retained a more epithelial-like morphology, with the effect being more pronounced at the 10 µM concentration.

## Supplementary Figure 3


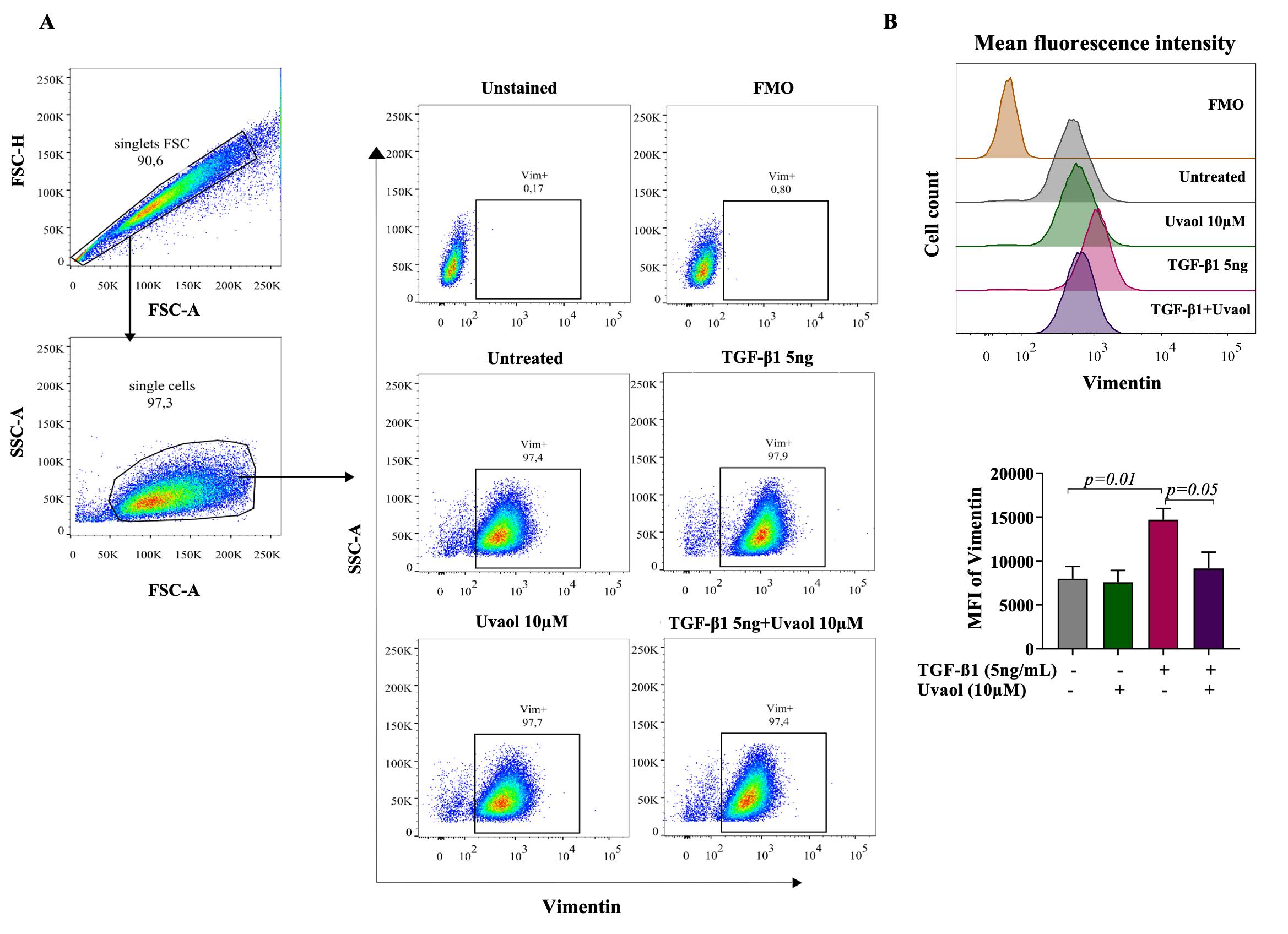


**Supplementary Figure** **S3**. Effect of uvaol on vimentin protein levels of EMT biomarker A549 cells were treated with TGF‐β1 with or without uvaol co‐treatment for 48 hr. Gate strategy employed for flow cytometric analysis. (A) The gating strategy for singlets is illustrated on a scatter plot of forward scatter height (FSC-H) versus forward scatter area (FSC-A). Subsequently (indicated by a black arrow), for singlet cells, we applied a gating strategy using side scatter area (SSC-A) versus FSC-A. (A) For the identification of cell populations, we used a gating strategy of SSC-A versus anti-Vimentin-PE. In (B), the histogram plot and bar chart represent the mean fluorescence intensity (MFI) of the total cells. Fluorescence minus one control (FMO) was used for gate identification of the positive cell population versus negative events. Data are from 4 independent experiments. In the graph bar, the groups were expressed as the means ± SEM. Graphs show statistical significance between groups, determined by one-way ANOVA followed by Tukey's post-test considering p < 0.05.

## Supplementary Figure 4


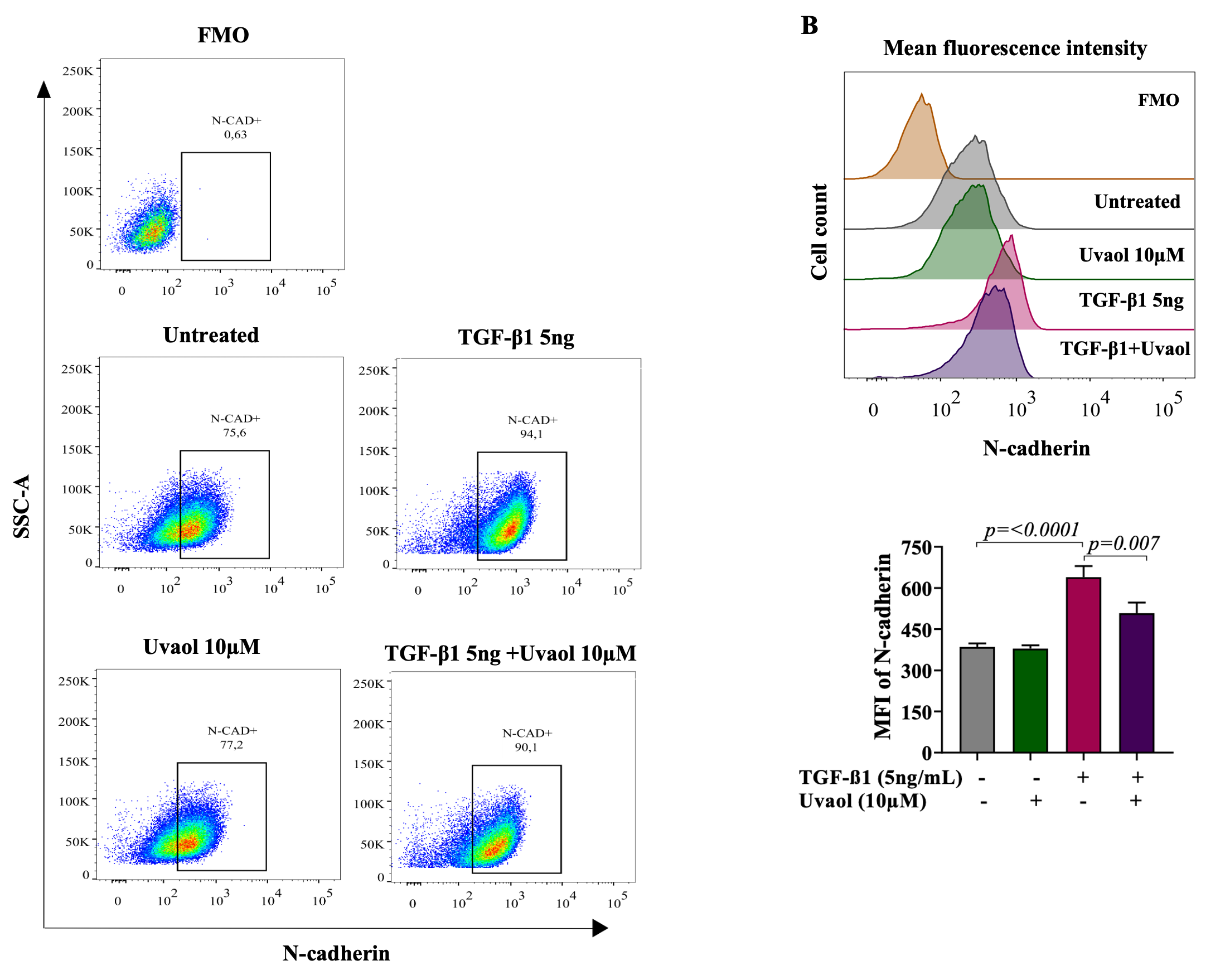


**Supplementary Figure S4.** Effect of uvaol on N-cadherin protein levels of EMT biomarker A549 cells were treated with TGF‐β1 with or without uvaol co‐treatment for 48 hr. (A) Gate strategy employed for flow cytometric analysis. In (B), the histogram plot and bar chart represent the mean fluorescence intensity (MFI) of total cells. Data are from 4 independent experiments. In the graph bar, the groups were expressed as the means ± SEM. Bar graphs show statistical significance between groups, determined by one-way ANOVA followed by Tukey's post-test considering p <0.05. Fluorescence minus one control (FMO) was used for gate identification of cell populations.

## Supplementary Figure 5


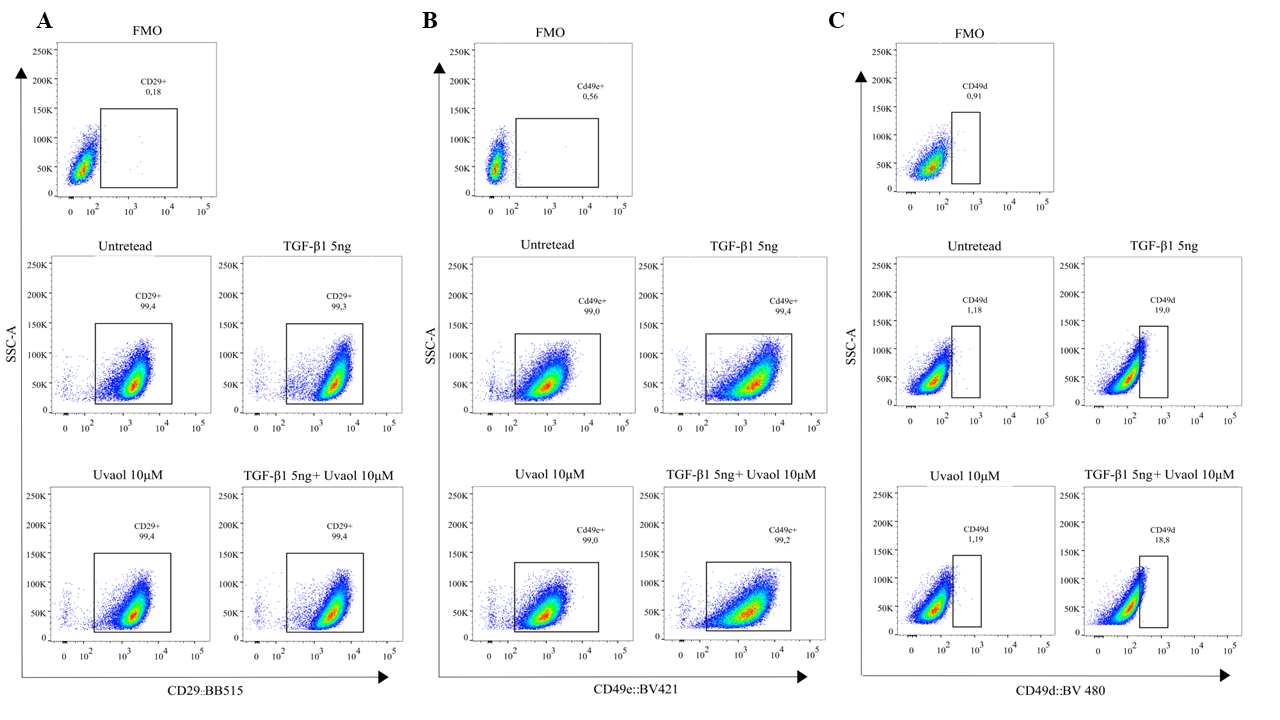


**Supplementary Figure S5.** Gate strategy employed for flow cytometric analysis for revealing the effect of uvaol on (**A)** CD29, **(B)** CD49e and **(C)** CD49d protein levels of EMT biomarker A549 cells were treated with TGF‐β1 with or without uvaol co‐treatment for 48 hr. Fluorescence minus one control (FMO) was used for gate identification of the cell population positive events.
